# Supplementary material for: Enhanced Adhesion of Electrospun Polycaprolactone Nanofibers to Plasma-Modified Polypropylene Fabric
Source: Polymers (Basel). 2023 Mar 28;15(7):1686. doi: 10.3390/polym15071686 (PMC10097108; doi:10.3390/polym15071686)
Supplement: Supplementary file 1 [file polymers-15-01686-s001.zip › polymers-2273948-SI.pdf]

Supplementary materials

Enhanced Adhesion of Electrospun Polycaprolactone Nanofibers to Plasma-modified Polypropylene Fabric

Lucie Janů[1]*, Eva Dvořáková[1], Kateřina Polášková[1,2], Martina Buchtelová[1], Petr Ryšánek[3], Zdeněk Chlup[4], Tomáš Kruml[4], Oleksandr Galmiz[5], David Nečas[1], Lenka Zajíčková[1,2,6]*

[1]*Plasma Technologies for Materials, Central European Institute of Technology - CEITEC, Brno University of Technology, Purkyňova 123, Brno, 612 00, Czech Republic*

[2]*Department of Condensed Matter Physics, Faculty of Science, Masaryk University, Kotlářská 2, 611 37 Brno, Czech Republic*

[3]*Faculty of Science, J.E. Purkyně University, 400 96 Ústí nad Labem, Pasteurova 15, Czech Republic*

[4]*Institute of Physics of Materials, The Czech Academy of Sciences, Žižkova 22, 616 00 Brno, Czech Republic*

[5]*Department of Physical Electronics, Faculty of Science, Masaryk University, Kotlářská 2, 611 37 Brno, Czech Republic*

[6]*Department of Theoretical and Experimental Electrical Engineering, Faculty of Electrical Engineering and Communication, Brno University of Technology, Technická 12, 616 00 Brno, Czech Republic*

* Correspondence: lucie.janu@ceitec.vutbr.cz (L.J.); lenkaz@physics.muni.cz (L.Z.)

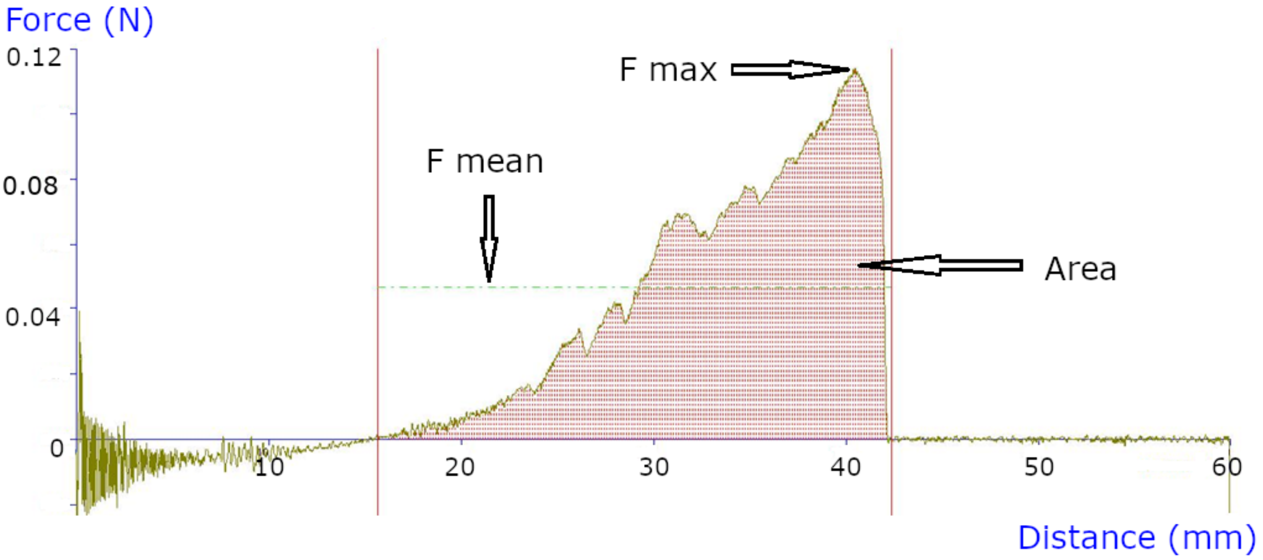

Figure S 1: Typical record of loop adhesion test measurement. The curve shown was obtained for the PP fabric treated with atmospheric PSJ in Ar.

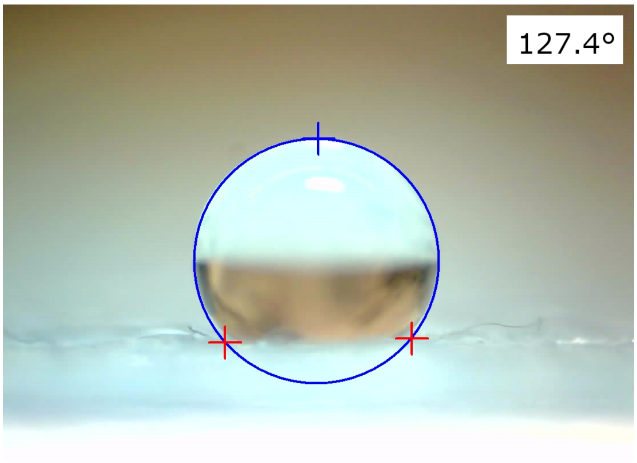

Figure S 2: WCA measurement – water droplet on reference PP fabric

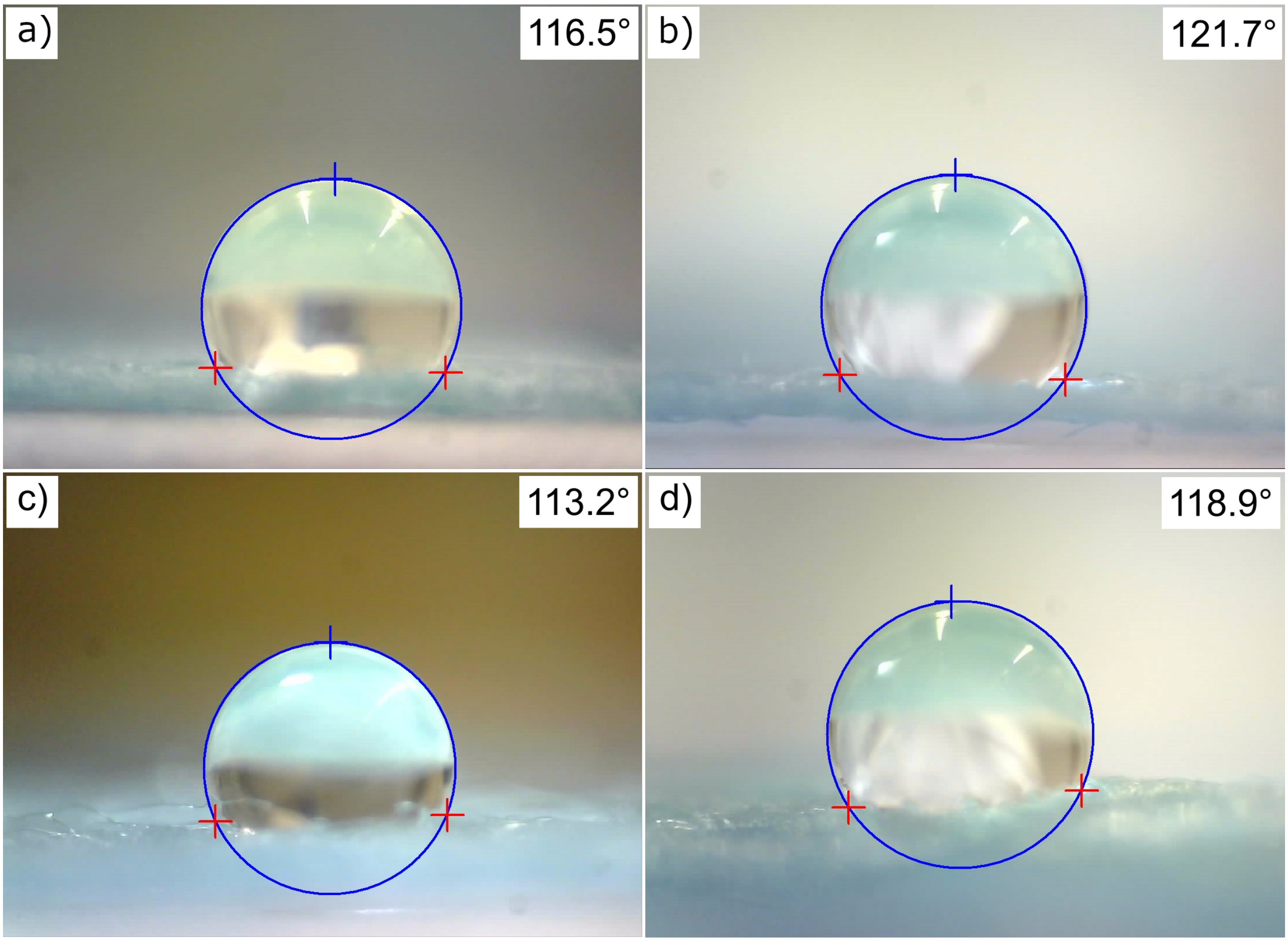

Figure S 3: WCA measurement of atmospheric PSJ treatment aging: examples of water droplets on Ar treated PP fabric day 0 (a) and day 15 (b) after treatment and on Ar+N_2 treated PP fabric day 0 (c) and day 15 (d) after treatment.

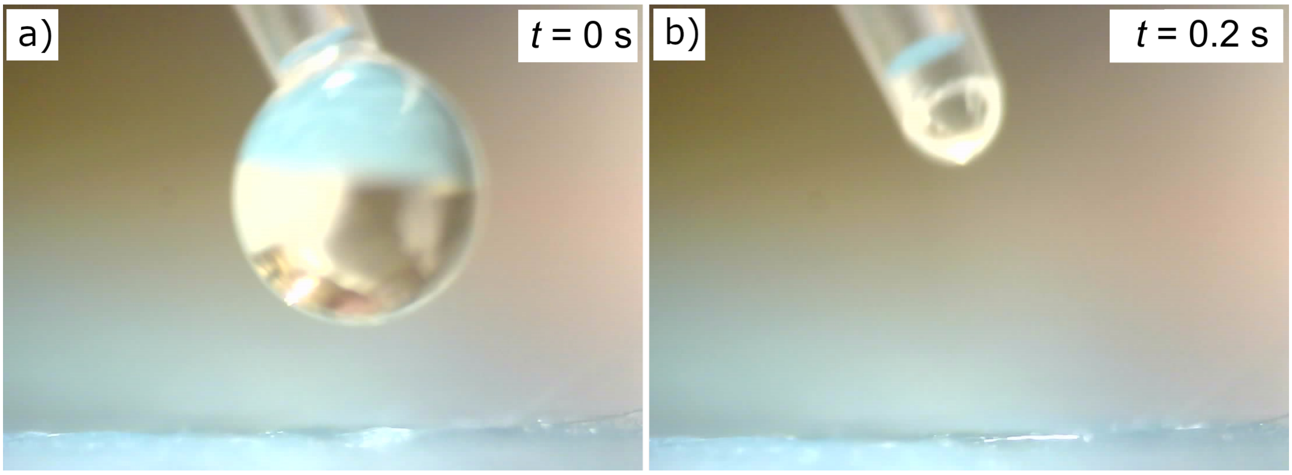

Figure S 4: Water droplet photographs documenting not measurable WCA on PP fabric modified by low-pressure plasma (example of O_2 treatment): droplet before placing on fabric (a), fabric immediately after droplet placing (b).
